# Clinical significance of the Kidney Donor Profile Index in deceased donors for prediction of post-transplant clinical outcomes: A multicenter cohort study

**DOI:** 10.1371/journal.pone.0205011

**Published:** 2018-10-05

**Authors:** Jong Hoon Lee, Woo Yeong Park, Young Soo Kim, Bum Soon Choi, Cheol Whee Park, Chul Woo Yang, Yong-Soo Kim, Kyubok Jin, Seungyeup Han, Byung Ha Chung

**Affiliations:** 1 Transplant Research Center, Seoul St. Mary's Hospital, Seoul, Korea; 2 Division of Nephrology, Department of Internal Medicine, Seoul St. Mary's Hospital, College of Medicine, The Catholic University of Korea, Seoul, Korea; 3 Department of Internal Medicine, Keimyung University School of Medicine, Daegu, Korea; 4 Keimyung University Kidney Institute, Daegu, Korea; 5 Division of Nephrology, Department of Internal Medicine, Uijeongbu St. Mary’s Hospital, College of Medicine, The Catholic University of Korea, Uijongbu, Korea; Universita degli Studi di Perugia, ITALY

## Abstract

**Background:**

We investigated whether the Kidney Donor Profile Index (KDPI) system is useful in predicting clinical outcomes in deceased donor kidney transplantation (DDKT).

**Methods:**

Four hundred sixty-nine kidney transplant recipients (KTRs) receiving kidneys from 359 deceased donors were included in this study, which involved three transplant centers. KTRs were divided into high and low KDPI KTR groups based on the median KDPI score of 67%. We compared clinical outcomes between the high KDPI and low KDPI groups.

**Results:**

There were no significant differences in the incidence of delayed graft function and acute rejection between high and low KDPI KTR groups. In comparison with histologic findings in allograft tissues obtained within three months from KT, the proportion of glomerulosclerosis was significantly higher in the high KDPI KTR group than in the low KDPI KTR group. With Kaplan-Meier analysis, the graft survival rate was significantly lower in the high KDPI KTR group than in the low KDPI KTR group (Log rank, P = 0.017), and multivariate analysis also demonstrated that a high KDPI score was a significant risk factor for death censored allograft failure (HR 2.62, 95% CI, 1.29–5.33, P = 0.008).

**Conclusion:**

The KDPI scoring system is useful in predicting allograft outcomes in a Korean DDKT cohort.

## Introduction

As the number of end stage renal disease (ESRD) patients increases, so does the demand for kidney donation [1]. However, a severe donor shortage results in the gradual prolongation of the waiting time for deceased donor kidney transplantation (DDKT) [2–5]. Thus, the use of kidneys from marginal donors, such as the elderly or those with renal impairment or underlying chronic kidney disease, has been proposed as an important strategy for solving this donor shortage [6–9]. The most widely used criteria for the definition of marginal donors has been the expanded criteria donor (ECD) according to the United Network for Organ Sharing (UNOS) [10]. Some previous studies have shown that the prognosis of kidney transplantation (KT) from ECD is not significantly different from that of KT from standard criteria donors (SCDs) [11, 12]. Therefore, the Kidney Donor Risk Index (KDRI) and Kidney Donor Profile Index (KDPI), which is the normalized percentile scores of KDRI, were developed as donor risk scoring systems [13, 14].

The KDPI incorporates more detailed donor factors than ECD criteria and has been validated according to the histologic status of the donor kidney and the donors’ age. [15]. Therefore, it has been expected to more precisely predict post-transplant allograft outcomes, and the validation of the KDPI would be more useful in discriminating the older donors. However, in contrast to ECD criteria, the clinical usefulness of KDPI has not been widely investigated in countries other than the United States (U.S.) [16]. Therefore, the value of the KDPI needs to be verified in each country before clinical application. In this regard, the aim of this study was to investigate the predictive value of KDPI for clinical outcomes using a DDKT cohort from multiple transplant centers in Korea.

## Materials and method

### Study population

Between October 1996 and May 2016, 509 cases of DDKT have been performed at three transplant centers (Seoul St. Mary’s Hospital, Uijeongbu St. Mary’s Hospital, and Keimyung University Hospital). Out of them, we included 469 kidney transplant recipients (KTRs) receiving kidneys from 359 deceased donors (DDs), in whom data for the calculation of KDPI were available. None of the transplant donors were from a vulnerable population and all donors or next of kin provided written informed consent that was freely given.

**Fig 1:**
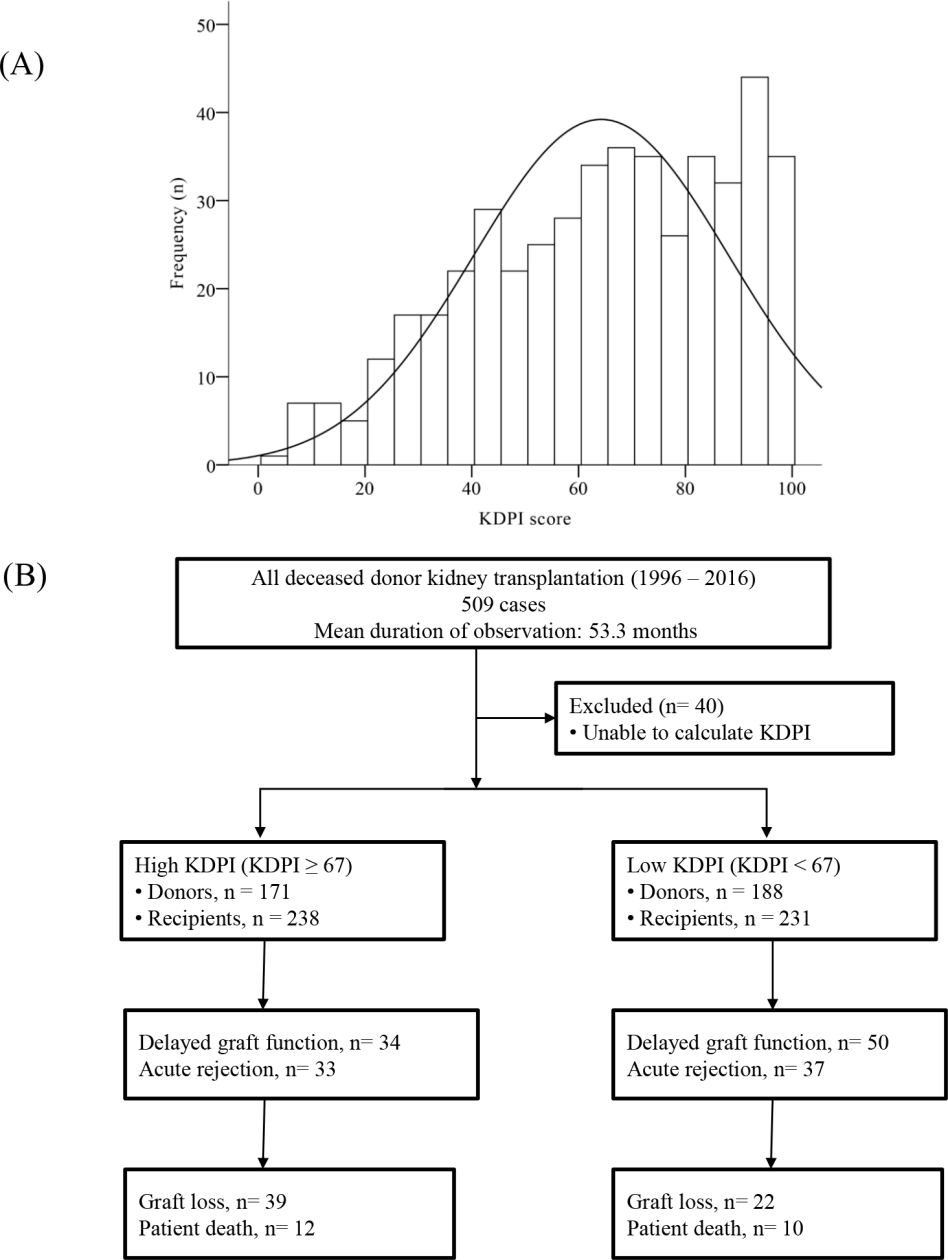
Distribution and algorithm. (A) Distribution of deceased donors by KDPI score. (B) Algorithm and distribution of kidney donors and recipients in this study. Patients were divided into high or low KDPI KTR groups based on the median value of KDPI of 67%. The incidence of acute rejection, delayed graft function, graft loss, and patient death was evaluated.

Donor data were collected, including age, height, weight, ethnicity, history of hypertension (HTN) or diabetes mellitus (DM), cause of death, serum creatinine, hepatitis C virus serology, and donation after cardiac death. The KDPI score was calculated with a “KDPI calculator” on the Organ Procurement and Transplantation Network (OPTN) website, using these 10 items [13, 14]. The distribution of KDPI scores is presented in Fig 1A. According to the calculated KDPI median of 67%, donors were divided into high KDPI (n = 171) and low KDPI (n = 188) donor groups. Hence, 238 recipients belonged to the high KDPI KTR group and 231 to the low KDPI KTR group (Fig 1B). Acute kidney injury (AKI) in DDs was diagnosed according to the Kidney Disease: Improving Global Outcomes (KDIGO) criteria as described in previous reports [17, 18].

### Clinical parameters and outcomes

The medical records of DDs and corresponding KTRs were retrospectively analyzed. Age, sex, body mass index (BMI–kg/m^2^), history of DM and HTN, cause of death, and estimated glomerular filtration rate (eGFR), an assessment of kidney function, from the day of admission to the day of KT were included in baseline donor data. In addition, the following baseline recipient data were collected: age, sex, BMI, history and duration of dialysis before KT, number of previous KTs, cause of ESRD, history of DM and HTN, cold ischemic time, number of human leukocyte antigen (HLA) mismatches, immunosuppressant type for induction, and maintenance and percentage of panel-reactive antibodies (PRAs).

The findings of allograft protocol biopsies performed at three months after KT in 246 KTRs were analyzed. The histologic grades of acute and chronic lesions in allograft tissue were compared in the high KDPI KTR group and low KDPI KTR group. The histologic evaluation was based on the Banff classification; evaluation items included glomerulitis (g), tubulitis (t), interstitial inflammation (i), and peritubular capillaritis (ptc) as acute lesions. The evaluation of chronic lesions included chronic glomerulopathy (cg), chronic tubular atrophy (ct), chronic interstitial fibrosis (ci), chronic intimal thickening (cv), arteriolar hyalionosis (ah), mesangial matrix increase (mm), and glomerulosclerosis (gs). Diagnosis of biopsy-proven acute rejection was made according to the Banff classification [19]. Delayed graft function (DGF) was defined as dialysis requirement within the first week after KT [20]. The death-censored allograft survival rate was defined as the time from KT to the commencement of an alternative renal replacement therapy, censored for death. Patient survival was defined as the time from KT until death from any cause. All missing data were managed by censoring since the last follow-up date.

The main objective of this study was to investigate the significance of high KDPI score on long-term allograft survival in DDKT. The primary outcome was long-term allograft survival rate in each KTR group. Secondary outcomes involved the incidence of DGF, the incidence of biopsy-proven acute rejection, the change of allograft function during the first year post-transplant (one, two, and three days; one and two weeks; and one, three, six, and 12 months after KT, as assessed by eGFR and calculated using the modification of diet in renal disease (MDRD) equation [21]), the chronic change in allograft tissue, and patient survival rate. To assess the risk of poor clinical outcomes when receiving a high KDPI kidney, allograft survival rate and patient survival rate were analyzed according to the baseline characteristics of recipients.

This study was approved by the institutional review boards of Seoul St. Mary’s Hospital (XC15RIMI0061K), Uijeongbu St. Mary’s Hospital (XC15RIMI0061U), and Keimyung University School of Medicine, Dongsan Medical Center (2017-08-019). The requirement for informed consent was waived because this study was a retrospective medical record review.

### Statistical analysis

Continuous variables with a normal distribution are presented as mean ± standard deviation (SD), whereas those with a non-normal distribution are presented as median value with a range of quadrants. Student’s t-test was used for analyzing continuous variables with a normal distribution, and the Mann-Whitney test was used for those with a non-normal distribution. Categorical variables are presented as count and percentage. The Chi-square test or Fisher’s exact test was used to analyze categorical variables. Kaplan-Meier curves and log-rank tests were utilized to describe and compare the death censored graft survival and patient survival rates. To define the risk factors that affected allograft and patient outcomes in the overall patient population, Cox proportional hazards regression analysis was used. As confounding variables for multivariable analysis of death censored allograft survival, the following factors were selected: recipient age [22], HLA mismatches [23], DM status [22], and re-transplant [23] and recipient BMI [23] based on previous studies. Spearman rank correlation analysis was used to analyze the relationship between KDPI scores and severity of glomerulosclerosis in allograft protocol biopsies. P-values <0.05 were considered statistically significant. All statistical analyses were performed using IBM SPSS Statistical software Version 21.0 (IBM, Armonk, NY, USA) and MedCalc Statistical software Version 15.5 (MedCalc Software, Ostend, Belgium).

## Results

### Comparison of baseline characteristics between high and low KDPI donor groups and between high and low KDPI recipient groups

In comparison with baseline characteristics, there were no significant differences based on sex, BMI, serum creatinine level, and death due to cerebrovascular accident between high and low KDPI donor groups. However, high KDPI donors were older and showed lower eGFR at organ procurement, with higher proportions of HTN and DM (P<0.001 for each). The proportion of donors with AKI was also higher in the high KDPI donor group than in the low KDPI donor group (P<0.001).

When comparing baseline characteristics of two recipient groups (high KDPI KTRs vs. low KDPI KTRs), mean age, the proportion of DM as the cause of ESRD, and BMI were significantly higher in the high KDPI KTR group than in the low KDPI KTR group (P<0.05 for all). There was no difference in the type of induction therapy, the proportion of KTRs with high PRA (>50%), and cold ischemic time between high and low KDPI KTR groups (Table 1).

**Table 1:** Comparison of clinical and laboratory parameters between high KDPI donor (or recipient) and low KDPI donor (or recipient).

**Variable**	**High KDPI** **(n = 188)**	**Low KDPI** **(n = 171)**	***P-*value**
**Donors**			
Age at KT (years)	54.7±8.7	35.7±12.5	<0.001
Gender (Male:Female)	112:76	135:36	0.197
BMI (kg/m^2^)	23.1±3.0	22.9±3.9	0.649
HTN, n (%)	70 (40.9%)	11 (5.9%)	<0.001
DM, n (%)	31 (18.1%)	2 (1.1%)	<0.001
Cause of donor death–CVA, n (%)	131 (76.6%)	138 (73.4%)	0.484
Baseline eGFR(ml/min/1.73m^2^)	64.5±30.6	78.7±49.3	<0.001
AKI, n (%)	111 (64.9%)	69 (36.7%)	<0.001
	**High KDPI** **(n = 238)**	**Low KDPI** **(n = 231)**	***P-*value**
**Recipient**			
Age at KT(year)	49.7±9.	47.2±10.0	0.006
Gender (Male:Female)	139:99	132:99	0.782
BMI (kg/m^2^)	23.2±3.	22.5±3.1	0.023
HTN, n (%)	207 (87%)	196 (84.8%)	0.508
DM, n (%)	61 (25.6%)	32 (13.9%)	0.001
Dialysis before KT, n (%)	236 (99.2%)	228 (98.7%)	0.629
Dialysis duration ^a^, years	7.4±10.9	8.0±4.8	0.394
Previous KT ^b^, n(%)	18 (7.6%)	29 (12.6%)	0.072
Cause of ESRD, n (%)			
Diabetes	51 (21.4%)	26 (11.3%)	0.003
Hypertension	54 (22.7%)	35 (15.2%)	0.037
GN	89 (37.4%)	107 (46.3%)	0.050
Others	44 (18.5%)	63 (27.3%)	0.023
Cold ischemic time (min)	244.5±113.9	246.4±135.8	0.874
HLA mismatch number	3.8±1.4	3.6±1.4	0.197
Induction, n (%)			
Basiliximab	172 (72.3%)	184 (79.7%)	0.062
ATG	66 (27.7%)	47 (20.3%)	0.062
Major immunosuppressant			
Tacrolimus: Cyclosporine	235:3	224:6	0.291
PRA > 50%, n (%)	30 (13.6%)	39 (19.8%)	0.091

Values are expressed as means ± SDs, n (%). eGFR is calculated using MDRD formula.

a. Duration of dialysis before transplantation

b. Recipient with previous kidney transplantation history.

KDPI, kidney donor profile index; KT, kidney transplantation; BMI, body mass index; HTN, hypertension; DM, diabetes mellitus; eGFR, estimated glomerular filtration rate; CVA; cerebrovascular accident; CKD, chronic kidney disease; AKI, acute kidney injury; ESRD, end-stage renal disease; GN, Glomerular nephritis; HLA, human leukocyte antigen; ATG, antithymocyte globulin; PRA, panel reactive antibody

### Comparison of the development of DGF, acute rejection, and allograft function within one year after KT between high and low KDPI KTR groups

**Fig 2:**
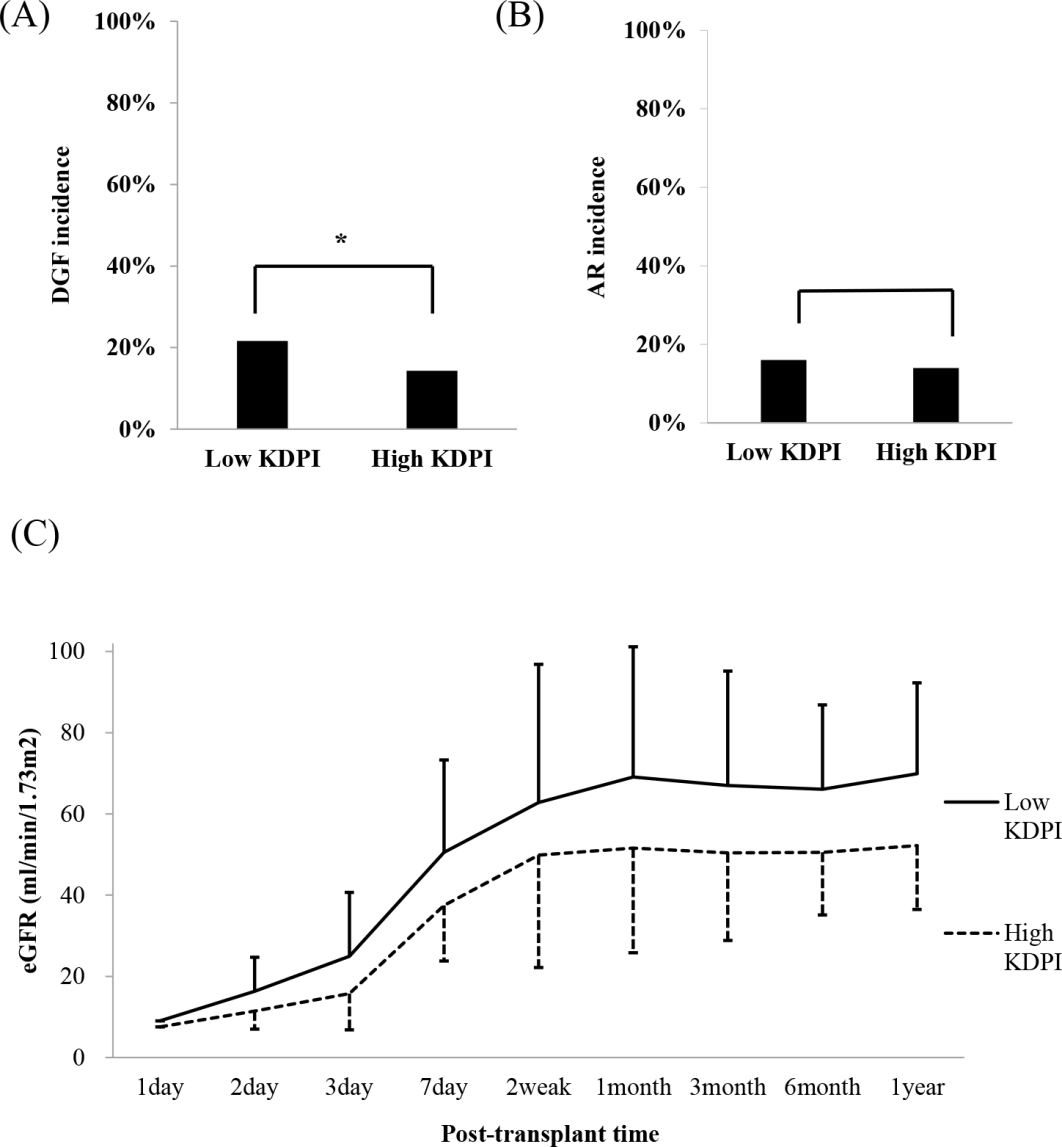
Analysis of short-term outcomes. (A) Comparison of the incidence of DGF between high and low KDPI KTR groups. *P<0.05 (B) Comparison of the incidence of biopsy-proven acute rejection between high and low KDPI KTR groups. (C) Comparison of the change of allograft function within one year after KT between high and low KDPI KTR groups (all P of each point <0.05).

The occurrence of DGF was significantly more frequent in the low KDPI KTR group than in the high KDPI KTR group (P<0.05) (Fig 2A). However, in multivariate analysis, it was not a significant factor in the development of DGF. The incidence of biopsy-proven acute rejection did not differ between the high KDPI KTR and low KDPI KTR groups (Fig 2B). Instead, acute rejection was correlated with recipient age (HR 0.970, CI 9.446–9.995, P = 0.017), immunologic factors including PRA (HR 1.011, CI 1.003–1.019, P = 0.010), and HLA mismatch number (HR 1.360, CI 1.107–1.673, P = 0.003). The high KDPI KTR group consistently showed deteriorated allograft function for one year after DDKT compared to the low KDPI KTR group (P<0.05 for all) (Fig 2C).

### Comparison of chronic injury scores in allograft tissues obtained within three months from KT between high and low KDPI KTR groups

**Fig 3:**
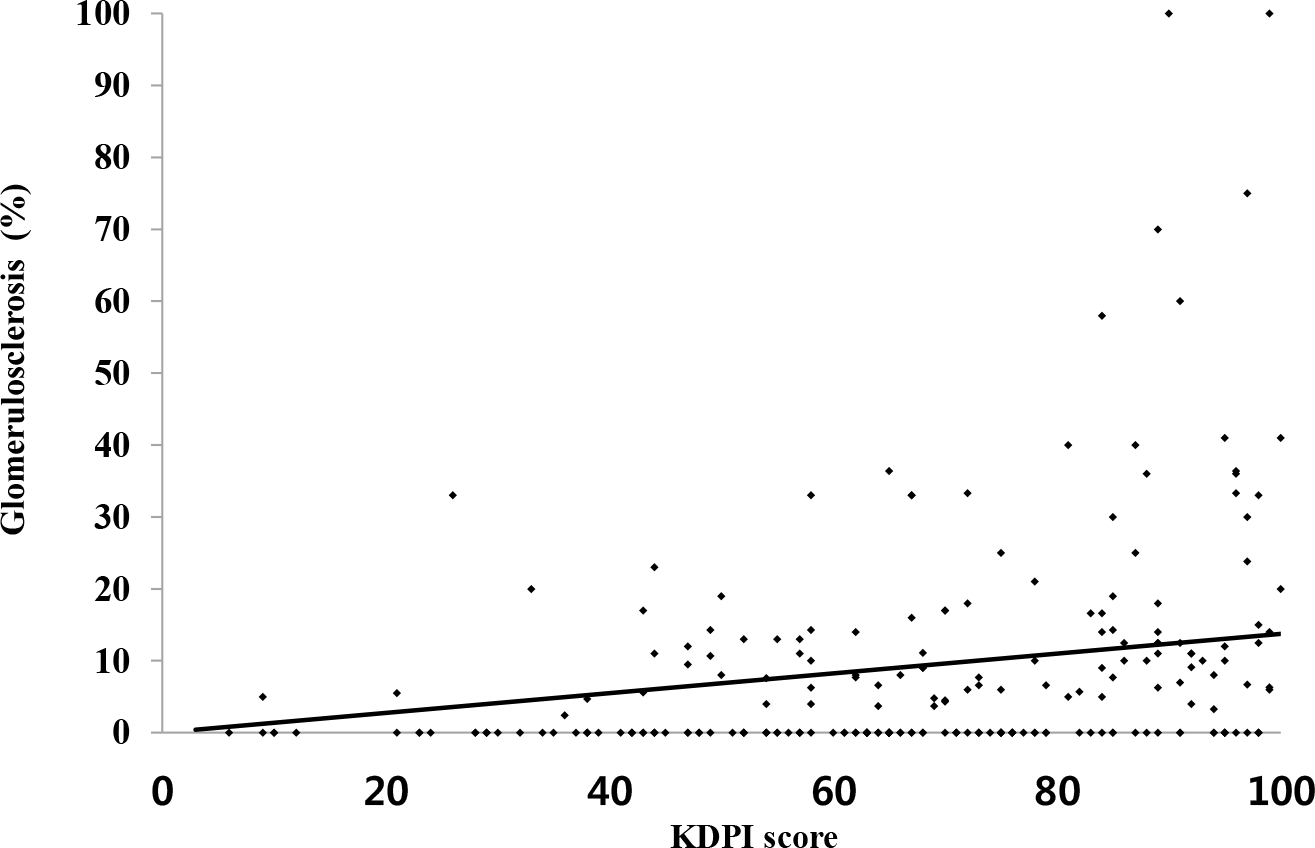
Relationship between KDPI score and glomerulosclerosis. Comparison of KDPI score and degree of chronic tissue injury (mean glomerulosclerosis percentage) in the results of allograft biopsy performed within three months after KT (rho = 0.368, P<0.001).

When chronic tissue injury markers such as glomerulosclerosis were compared in allograft tissue obtained within three months from KT, the percentages of total glomerulosclerosis and global glomerulosclerosis were significantly higher in the high KDPI KTR group than in the low KDPI KTR group (P<0.001 for both) (Table 2). The glomerulosclerosis was significantly associated with the KDPI score (rho = 0.368, P<0.001) (Fig 3). Other histologic findings, such as cg, ct, ct, cv, ah and mm scores, did not differ between the two groups.

**Table 2:** Comparison of chronic injury scores in allograft tissues obtained within three months from KT between high and low KDPI KTR groups.

Banff qualifier code	High KDPI (n = 129)	Low KDPI (n = 117)	*P*-value
Chronic glomerulopathy (cg)	0.01±0.09	0.03±0.28	0.928
Chronic tubular atrophy (ct)	0.40±0.58	0.27±0.47	0.088
Chronic interstitial fibrosis (ci)	0.40±0.60	0.29±0.49	0.157
Chronic intimal thickening (cv)	0.06±0.29	0.02±0.13	0.315
Arteriolar hyalinosis (ah)	0.04±0.24	0.12±0.41	0.088
Mesangial matrix increase (mm)	0.18±0.39	0.23±0.51	0.823
Global glomerulosclerosis (%)	11.61±17.65	3.32±7.36	<0.001
Segmental glomerulosclerosis (%)	1.10±5.39	0.44±1.93	0.787
Glomerulosclerosis (%)	12.67±18.67	3.80±7.37	<0.001

Note: Mann-Whitney U test is used for comparison of histologic grade.

### Comparison of the death censored allograft survival rate between high KDPI KTR and low KDPI KTR groups

**Fig 4:**
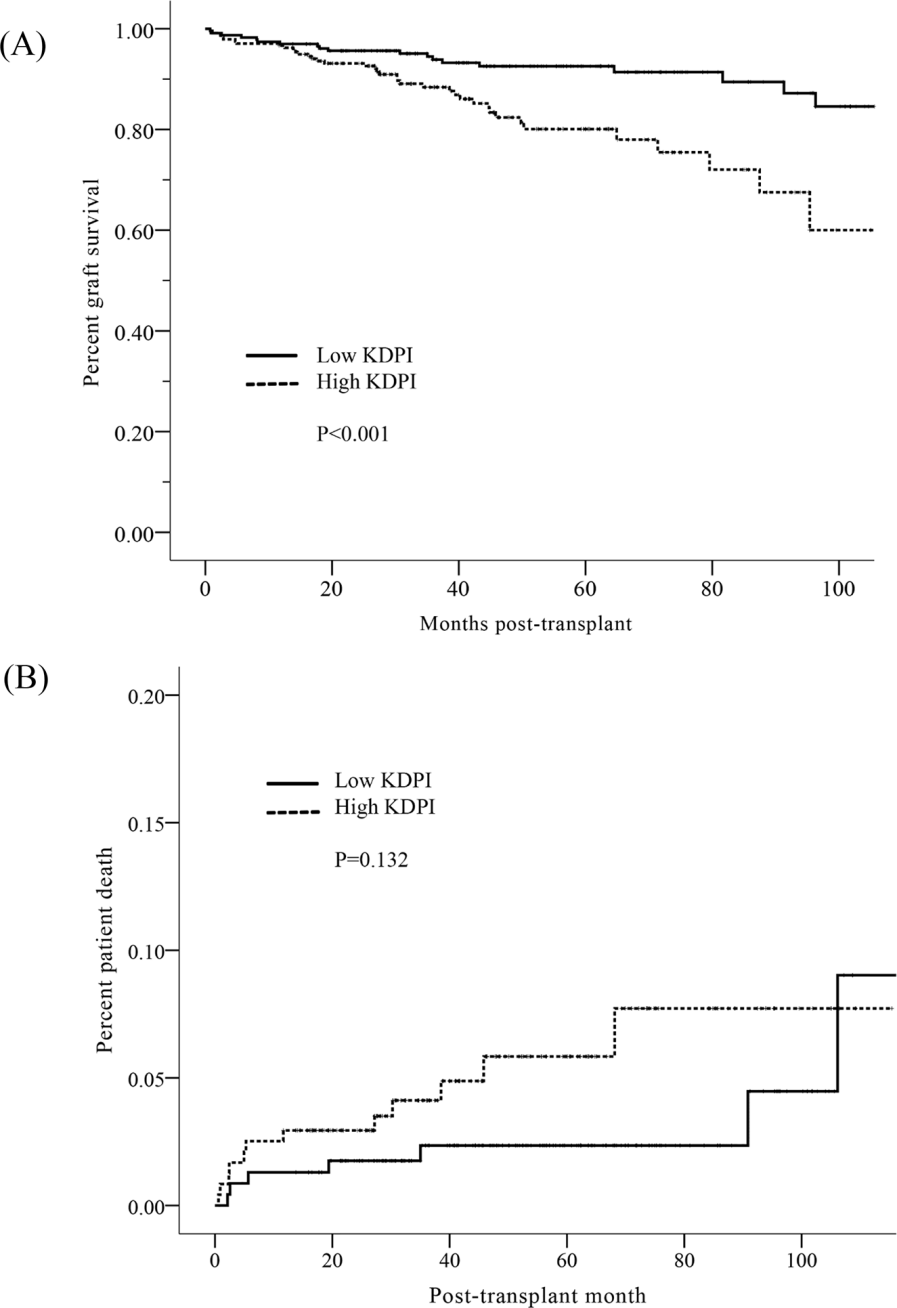
Analysis of long-term outcome. (A) Comparison of death-censored graft survival rates between high and low KDPI KTR groups (P<0.001, Log-rank test) (B) Comparison of patient survival rates between high and low KDPI KTR groups (P = 0.132, Log-rank test).

A total of 61 cases of allograft failure developed during the follow-up period. Of these, 39 developed in the high KDPI KTR group, and 22 developed in the low KDPI KTR group. As a cause of graft failure, chronic rejection occurred more frequently in the high KDPI KTR group than in the low KDPI KTR group. However, there were no differences in the incidence of other causes including acute rejection, recurrent GN, ischemia, infection, and BK virus-associated nephropathy between the two groups (Table 3). The low KDPI KTR group exhibited a significantly better allograft survival rate than the high KDPI KTR group (P<0.001, Log-rank test) (Fig 4A). High KDPI KT was a significant risk factor for allograft failure in a univariate model (HR 3.050, CI 1.577–5.896, P<0.001) and also in the multivariate model (adjusted HR 3.695, CI 1.873–7.288, P<0.001) by Cox regression analysis adjusted for recipient age, DM, recipient BMI, re-transplant, and HLA mismatch number, and a KDPI score was also identified as a significant risk factor for graft failure (Table 4). A total of 22 cases of patient death occurred during the follow-up period. Twelve patients in the high KDPI KTR group died, as did 10 in the low KDPI KTR group. Survival rates were not significantly different between high and low KDPI KTR groups (Fig 4B). In addition, cause of death did not differ between the two groups (Table 5).

**Table 3:** Comparison of death censored graft failure of high and low KDPI recipient group.

Variable	High KDPI (n = 238)	Low KDPI (n = 231)	*P-*value
Total graft failure, n (%)	39 (16.4%)	22 (9.5%)	<0.001
Causes of graft failure, n (%)			
Acute rejection	11 (4.6%)	8 (3.5%)	0.137
Chronic rejection	9 (3.8%)	1 (0.4%)	0.014
Recurrent GN	4 (1.7%)	1 (0.4%)	0.058
Ischemia	0 (0%)	3 (1.3%)	0.418
Infection	2 (0.8%)	1 (0.4%)	0.503
BKVAN	2 (0.8%)	1 (0.4%)	0.272

The difference in the incidence of graft failure due to each cause was analyzed using binary Cox regression analysis.

GN, Glomerular nephritis; BKVAN, BK virus associated nephropathy

**Table 4:** Association between KDPI score, dichotomy by median KDPI and death censored graft failure by Cox regression modeling.

	HR	95% CI	*P-*value
**Univariate model**			
High KDPI (dichotomy)	3.050	1.577–5.896	<0.001
KDPI score	1.020	1.005–1.035	0.008
**Multivariate model** ^a^			
High KDPI (dichotomy)	3.695	1.873–7.288	<0.001
KDPI score	1.025	1.009–1.041	0.002

a. Adjusted by recipient age, recipient diabetes, recipient body mass index, previous kidney transplantation history, HLA mismatch number

**Table 5:** Comparison of the cause of patient death between high and low KDPI KTR group.

	High KDPI (n = 238)	Low KDPI (n = 231)	*P-*value
Total death, n (%)	12 (5.0%)	10 (4.3%)	0.132
Causes of death, n (%)			
CAD	3 (1.3%)	1 (0.4%)	0.380
CVA	1 (0.4%)	0	0.613
Infection	3 (1.3%)	4 (1.7%)	0.743
Malignancy	1 (0.4%)	0	0.589
Others ^a^	2 (0.8%)	1 (0.4%)	0.467
Unknown	2 (0.8%)	4 (1.7%)	0.572

The difference in the incidence of patient death due to each cause was analyzed using binary Cox regression analysis.

a. Hepatic failure and heart failure in high KDPI group, GI bleeding in low KDPI group

CAD, Coronary artery disease; CVA, Cerebrovascular accident

## Discussion

Assessment of donor quality is an important issue in DDKT for deciding whether to discard the kidney and for predicting allograft outcomes after KT. Results of this study demonstrated that classification by KDPI had a significant predictive value for graft survival, which suggests that KDPI can be a useful tool for predicting outcomes in clinical practice in Korea.

At first, investigators examined the distribution of KDPI scores. It was necessary to establish high risk donors for allocation. A donor with a KDPI score of 85 or more is considered high risk in the U.S. This concept has supplanted the ECD/SCD dichotomy in the U.S. kidney allocation system [24]. However, it is controversial whether to use the same reference point of 85 in other nations. The KDPI can be estimated to be low or high by several factors such as race and the medical environment of various nations and transplant centers. This study did not include KTs involving African American donors, donors with HCV, and cardiac death donors. In these circumstances, the KDPI can be underestimated. These factors might lead one to believe that donors in this study were superior to other donors according to the applied KDPI mapping system. In fact, when survey subjects were divided by a KDPI of 85, 31.5% of ECD donors were included in the low KDPI group. Therefore, the median KDPI score of the group was set as a reference value. More research is needed on the definition of high risk groups for the DD allocation system. Coordination is also required at the national level and/or the transplant center where the KDPI is applied.

For the above reasons, the median value was used as criterion in this study. Since the KDPI score is represented only as an integer and there are patients with same KDPI score, dividing donor by the median value did not allocate the same number of donors to the high/low KDPI group and caused an imbalance.

In predicting short-term clinical outcomes, cross tab analysis revealed that the incidence of DGF was higher in the group with low KDPI (Fig 2A). However, multivariate regression analysis showed no significant results. In KTRs with a history of KT, the incidence of DGF increases [25]. In our study, DGF incidence was significantly higher in recipients with previous kidney transplantation history than those without history (HR = 2.391, CI 1.229–4.652, P = 0.010). The current analysis of basic characteristics revealed that the repeated KT recipient was biased toward the lower KDPI group (Table 1). For this reason, this study is presumed to have an increased incidence of DGF in the low KDPI group because the influence of recipient factor was greater than the donor factor. Since DGF is caused by ischemic injury [26], only donor creatinine level and donors in AKI status were associated with an increase of DGF in the current study.

Allograft function was significantly lower in the high KDPI group than in the low KDPI group for one year post-KT. It may be that the capacity for recovery in allograft kidneys from high KDPI donors is lower than those from low KDPI donors. Indeed, the baseline eGFR was lower in high KDPI donors than in low KDPI donors. In addition, allograft biopsy obtained within three months of KT demonstrated significantly higher glomerulosclerosis scores in the high KDPI group. Moreover, KDPI score had a significant correlation with the degree of glomerulosclerosis. Considering the short interval post-KT, chronic findings of allograft biopsy may reflect the unique chronic histologic damage of the donor, rather than damage developed after KT. As the degree of glomerulosclerosis in allograft kidneys is associated with graft failure after KT [27, 28], The specific reflection of KDPI on glomerulosclerosis is considered to be a cause of strong predictive power for allograft survival.

In previous studies, ECD classification could not predict allograft outcomes, which suggests the limitation of a dichotomous classification system. [29, 30]. ECD classification failed to predict allograft survival when applied to data (P = 0.248, Log-rank test) (S1 Fig). This suggests that the ECD versus SCD dichotomy is not a detailed tool to predict post-transplant clinical outcomes in DDKT, perhaps because the ECD group consisted of heterogeneous patients in terms of risk for adverse allograft outcomes.

In contrast, KDPI had a significant predictive power for graft failure, and this finding was confirmed by the dichotomy based on the median value and the KDPI score. This result suggests that the risk for allograft failure may show a linear relationship to KDPI score in DDs. When the causes of graft failure in groups divided by KDPI were analyzed, although biopsy-proven acute rejection was the most common cause of graft failure in both groups, there was no significant difference between the two groups. The incidence of chronic rejection, which is the second leading cause of graft failure in the high KDPI KTR group, occurred significantly more often in the high KDPI KTR group than in the low KDPI KTR group. There may be controversy in the interpretation of this result. As seen in our data, the glomerulosclerosis was significantly associated with the KDPI score. The degree of glomerulosclerosis can also be evaluated as evidence of chronic rejection [31, 32]. Because of the possible consequences of these differences in interpretation of glomerulosclerosis, further studies are needed to confirm whether the incidence of chronic rejection increases as the quality of donor kidneys decreases.

This study had some limitations. First, this study calculated retrograde KDPI values from patients who had undergone transplantation and found a correlation with prognosis. Originally, KDPI is a prospective predictor, and this retrospective study has the potential to interfere with confounding factors. For example, low KDPI donor kidney may have a good prognosis due to the relatively young recipients not only due to quality of donor. And prognosis of high KDPI donor may be better than the actual one, since clinicians may have selectively performed DDKT with good quality of kidney. Therefore, a prospective study is needed to confirm the predictive power of KDPI. Second, this study did not provide a cut-off value to be associated with poor prognosis in terms of allograft failure. However, this scoring system can be used to predict post-transplant clinical outcomes and also to select recipients who can demonstrate relatively favorable clinical outcomes. Third, the KDPI is a concept of consecutive percentiles, but this study did not demonstrate a correlation between prognosis and difference in interval. Finally, because allograft biopsies were not performed in all KTRs, histologic findings of allografts did not reflect the prognosis of all KTRs, and it is unclear whether chronic changes in allograft biopsies can reflect long-term allograft outcome.

In conclusion, the KDPI scoring system is useful in predicting allograft outcomes in a Korean DDKT cohort. The results suggest that, although the mapping and the items of KDPI were created based on U.S. data, they can be used as tools for quality evaluation of donors even in other nations and medical environments.

## Supporting information

S1 Fig Analysis of graft failure based on ECD classification. Comparison of death-censored graft survival rates between ECD-KT and SCD-KT groups (P = 0.248, Log-rank test).
(TIF)

S1 Dataset Database. (XLSX)
