# Supplementary figures and images for: Clinical significance of the Kidney Donor Profile Index in deceased donors for prediction of post-transplant clinical outcomes: A multicenter cohort study
Source: PLoS One. 2018 Oct 5;13(10):e0205011. doi: 10.1371/journal.pone.0205011 (PMC6173429; doi:10.1371/journal.pone.0205011)

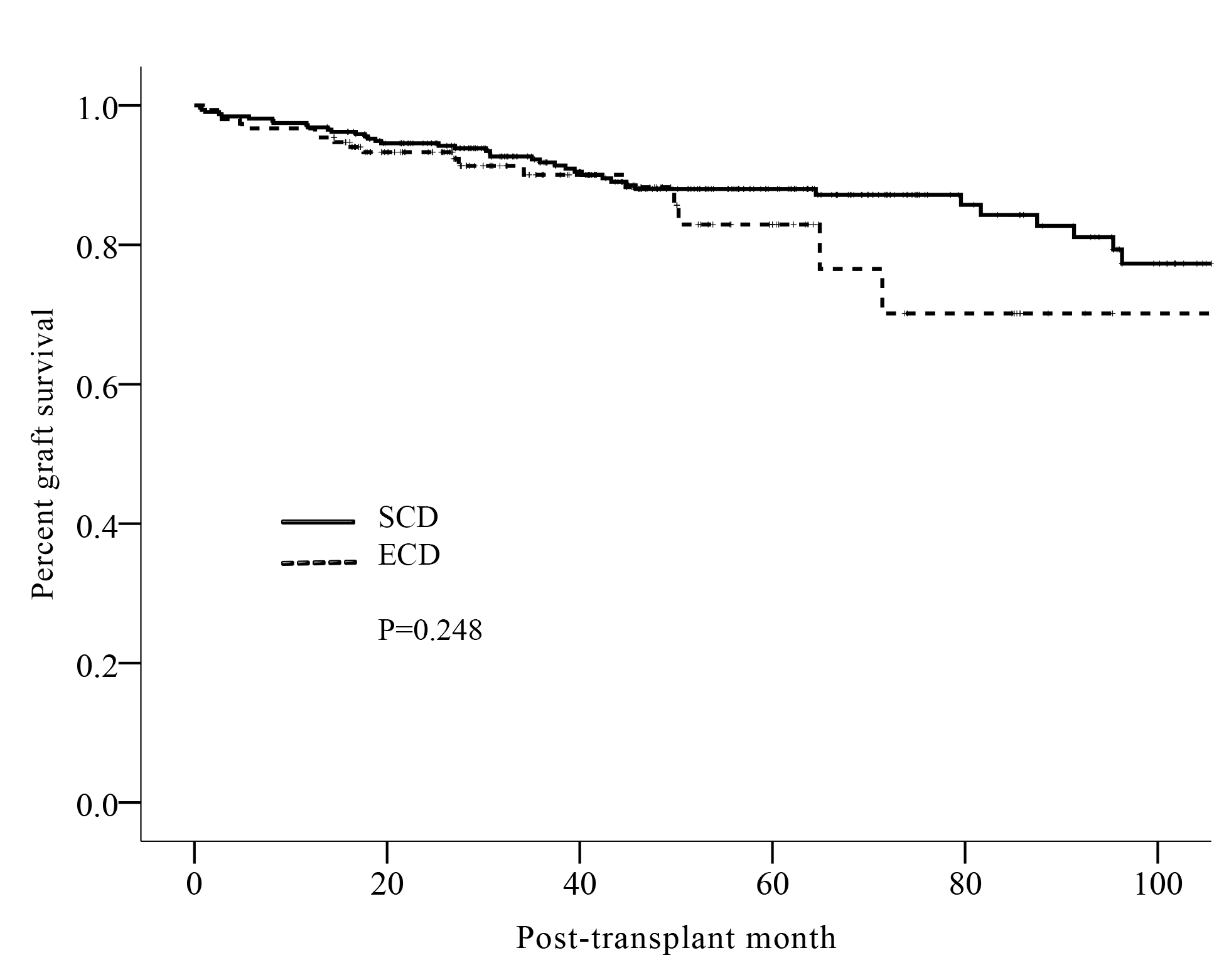

Supplement: S1 Fig — Comparison of death-censored graft survival rates between ECD-KT and SCD-KT groups (P = 0.248, Log-rank test). (TIF) [file pone.0205011.s001.tif]
